# Standards, Options and Recommendations for the use of appetite stimulants in oncology (2000)

**DOI:** 10.1038/sj.bjc.6601090

**Published:** 2003-08-15

**Authors:** J C Desport, G Gory-Delabaere, M P Blanc-Vincent, P Bachmann, J Béal, R Benamouzig, V Colomb, D Kere, J C Melchior, G Nitenberg, B Raynard, S Schneider, P Senesse

**Affiliations:** 1 CHU Dupuytren, Limoges, France; 2 FNCLCC, Paris, France; 3 Centre Léon Bérard, Lyon, France; 4 Centre Oscar Lambret, Lille, France; 5 Hôpital Avicenne, Bobigny, France; 6 Hôpital Necker, Paris, France; 7 Centre Val d'Aurelle, Montpellier, France; 8 Hôpital Raymond Poincaré, Garches, France; 9 Institut Gustave Roussy, Villejuif, France; 10 Hôpital Antoine Béclère, Clamart, France; 11 Hôpital de l'Archet, Nice, France

**Keywords:** neoplasms complications, appetite stimulants, practice guideline

Anorexia and cachexia are serious complications frequently found in patients with cancer (Bozetti, 1995; Donnelly and Walsh, 1995). They are present in about 10% of patients at the time of diagnosis (Bozetti *et al*, 1989). Multiple factors are involved in their aetiology (Puccio and Nathanson, 1997). The resultant malnutrition is associated with a poorer response to anticancer treatment and an impaired quality of life (Holmes and Dickerson, 1987; Bozetti, 1995; De Conno *et al*, 1998). Many clinical trials have been undertaken to evaluate the efficacy of drugs thought to be appetite stimulants.

## OBJECTIVES

The objective of these guidelines is to define which drugs have a certain or probable appetite-stimulating effect, which ones have no demonstrated effect and to describe any adverse effects in adult patients with cancer. These guidelines do not cover specific clinical cancer situations in which these drugs must be used.

## METHODS

The details of the full methodology have been previously published (Fervers *et al*, 2001). In summary, a multidisciplinary working group was set up by the French National Federation of Cancer Centres (Fédération Nationale des Centres de Lutte Contre le Cancer–FNCLCC) to review the literature on the use of appetite stimulants in oncology.

A literature search was performed in four database: *Medline*® (January 1990–June 1999), *Cancerlit*® (January 1990–April 1999), *Embase*® (January 1990–July 1999) and the *Cochrane Library*® (1999, issue 2). The following key words were used: appetite stimulants or anorexia/drug therapy or cachexia/drug therapy or appetite associated with neoplasms. The list of references thus identified was completed by the members of the working group with pertinent references from their personal bibliographic databases.

A total of 55 reports of randomised clinical trials, published in English or French, evaluating the appetite-stimulating effect of corticosteroids, synthetic progestogens and other drugs in cancer patients were selected for these guidelines. Since appetite stimulants are used to increase appetite in patients, this was the primary outcome used in the analysis of the results in these trials. The secondary outcomes were: improved quality of life; increase in body weight; increased food consumption; decrease in nausea and/or vomiting and improvement of anthropometric and biological parameters. In the absence of the primary outcome (increased appetite), none of the secondary outcomes are considered sufficient to confirm an appetite stimulating effect.

After selection and critical appraisal of this literature, the working group defined the ‘Standards’, ‘Options’ and ‘Recommendations’ (SOR) for the use of appetite stimulants in patients with cancer, based on a synthesis of the best available evidence.

‘Standards’ identify clinical situations for which there exist strong indications or contraindications for a particular intervention and ‘Options’ identify situations for which there are several alternatives, none of which have shown clear superiority over the others (Table 1

**Table 1:** Definition of Standards, Options and Recommendations

Standards	Procedures or treatments that are considered to be of benefit, inappropriate or harmful by unanimous decision, based on the best available evidence
	
Options	Procedures or treatments that are considered to be of benefit, inappropriate or harmful by a majority, based on the best available evidence
	
Recommendations	Additional information to enable the available options to be ranked using explicit criteria (e.g. survival, toxicity) with an indication of the level of evidence

). In any SOR, there can be several ‘Options’ for a given clinical situation. ‘Recommendations’ enable the ‘Options’ to be weighted according to the available evidence. Several interventions can be recommended for the same clinical situation, so that clinicians can make a choice according to specific clinical parameters, for example, local circumstances, skills, equipment, resources and patient preferences. The adaptation of the SOR to the local situation is allowable if the reason for the choice is sufficiently transparent and this is crucial for successful implementation. Inclusion of patients in clinical trials is an appropriate form of patient management in oncology and is recommended frequently within the SORs, particularly in situations where evidence is too weak to support an intervention.

The type of evidence underlying any ‘Standard’, ‘Option’ or ‘Recommendation’ is indicated using a classification developed by the FNCLCC based on previously published models. The level of evidence depends not only on the type and quality of the studies reviewed, but also on the concordance of the results (Table 2

**Table 2:** Definition of level of evidence

*Level A*
There exist a high-standard meta-analysis or several high-standard randomised clinical trials which give consistent results

*Level B*
There exist good quality evidence from randomised trials (B1) or prospective or retrospective studies (B2). The results are consistent when considered together

*Level C*
The methodology of the available studies is weak or their results are not consistent when considered together

*Level D*
Either the scientific data do not exist or there is only a series of cases

*Expert agreement*
The data do not exist for the method concerned, but the experts are unanimous in their judgement

). When no clear scientific evidence exists, judgement is made according to the professional experience and consensus of the expert group (‘expert agreement’).

These guidelines were then reviewed by a group of independent experts (see the Appendix) and finalised after taking into consideration their comments. This summary version is based on an integral version that was validated and published in 2000 (Desport *et al*, 2000) and is also available at: http://www.fnclcc.fr. These guidelines will be updated when new scientific data become available or when there is a change in expert agreement.

### Corticosteroids

Corticosteroids are appetite stimulants (level of evidence: B1). There is insufficient information available to define the optimal dose and scheduling for their use in this indication (recommendation).

### Synthetic progestogens

#### Megesterol acetate

Megesterol acetate is an appetite stimulant (level of evidence: B1). It results in a significant increase in appetite and there is a beneficial effect on body weight in patients with cancer (standard, level of evidence: B1). The minimum efficacious dose is 160 mg day^−1^ (level of evidence: B1). If there is no response, 480 mg day^−1^, which seems to be the optimal dose, can be used (recommendation, level of evidence: C). There is no evidence that doses greater than 480 mg day^−1^ have a higher efficacy (level of evidence: B1).

#### Medroxyprogesterone acetate (MPA)

Medroxyprogesterone acetate is an appetite stimulant (level of evidence: B1). It results in a significant increase in appetite (level of evidence: B1). The effect on weight gain has not been confirmed (level of evidence: C). Randomised clinical trials should be undertaken to investigate the optimal dose and duration of administration of this drug, although the minimum dose, shown to have a positive effect on appetite in published trials was 200 mg day^−1^ (recommendation, expert agreement).

### Other drugs

Cyproheptadine may be an appetite stimulant, but adverse effects have been reported (level of evidence: C). Dronabinol, metoclopramide, nandrolone and pentoxifylline have not been shown to have any appetite-stimulating effects (level of evidence: C). These drugs should not be used outside the setting of a randomised clinical trial (recommendation, expert agreement). Hydrazine sulphate is not an appetite stimulant (level of evidence: A).

### Management strategy: appetite stimulants for use in patients with cancer

Corticosteroids (no French product licence), megestrol acetate (no French product licence) and MPA (French product licence) can be used in the treatment of anorexia and weight loss in patients with cancer (recommendation, level of evidence: B1). Appetite stimulants can be used in combination with or after failure of dietetic and oral nutritional management (recommendation, expert agreement).

The use of appetite stimulants is particularly warranted in patients with incurable disease (recommendation, level of evidence: C). Appetite stimulants can be administered to patients with any type of tumour (recommendation, expert agreement). The optimal mode of administration for these products is not known. Hydrazine sulphate should not be used (standard, level of evidence: A).

Cyproheptadine, dronabinol, metoclopramide, nandrolone and pentoxifylline should only be used in the setting of a randomised clinical trial (standard, expert agreement).

## Appendix

### Reviewers

F Arnaud-Battandier (Nestlé Clinical Nutrition, Sèvres, France), L Battel-Copel (Institut Curie, Paris, France), RJ Bensadoun (Centre Antoine Lacassagne, Nice, France), JJ Body (Institut Jules Bordet, Bruxelles, Belgium), P Bourget (Institut Gustave Roussy, Villejuif, France), E Bruera (Edmonton General Hospital, Alberta, Canada),R Brunet (Institut Bergonié, Bordeaux, France), P Canal (Institut Claudius Regaud, Toulouse, France), T Conroy (Centre Alexis Vautrin, Vandœuvre-lès-Nancy, Nancy, France), JY Coquin (Institut Paoli-Calmettes, Marseille, France), S Culine (Centre Val d’Aurelle, Montpellier, France), D Cupissol (Centre Val d’Aurelle, Montpellier, France), J Delarue (CHU Bretonneau, Tours, France), M Ducreux (Institut Gustave Roussy, Villejuif, France), MN Fallewee (Centre Antoine Lacassagne, Nice, France), P Fargeot (Centre Georges-François Leclerc, Dijon, France), M Ferry (Centre Hospitalier, Valence, France), G Freyer (CHU Lyon Sud, Pierre Bénite, France), G Ganem (Le Mans, France), A Giacosa (IST National Cancer Institute, Geneva, Italy), MC Gouttebel (CH, Roanne, France), C Guedon (Hôpital Charles Nicolle, Rouen, France), X Hebuterne (Hôpital de L’Archet, Nice, France), P Haegele (Centre Paul Strauss, Strasbourg, France), S Hoffstetter (Centre Alexis Vautrin, Vandœuvre-lès-Nancy, Nancy, France), MC Husson (CNIMH, Paris, France), I Krakowski (Centre Alexis Vautrin, Vandœuvre-lès-Nancy, Nancy, France), F Lakdja (Institut Bergonié, Bordeaux, France), JF Latour (Centre Léon Bérard, Lyon, France), I Madelaine (Hôpital St Louis, Paris, France), Y Merrouche (CHRU, Saint-Etienne, Y France), F Montange (Centre Alexis Vautrin, Vandœuvre-lès-Nancy, Nancy, France), C Pichard (Hôpital universitaire de Genève, Genève, Switzerland), F Pinguet (Centre Val d'Aurelle, Montpellier, France), P Rebattu (Centre Léon Bérard, Lyon, France), D Rigaud (Hôpital Bichat, Paris, France), P Rougier (CHU Ambroise Paré Boulogne, France), P Ruffié (Institut Gustave Roussy, Villejuif, France), Mme Salamagne (Hôpital Paul Brousse, Villejuif, France), M Schneider (Centre Antoine Lacassagne, Nice, France), JP Sculier (Institut Jules Bordet, Bruxelles, Belgium), M Simon (Centre Alexis Vautrin, Vandœuvre-lès-Nancy, Nancy, France), N Tubiana (CHU Dupuytren, Limoges, France), B Vellas (CHU Purpan, Toulouse, France), and B Weber (Centre Alexis Vautrin, Vandœuvre-lès-Nancy, Nancy, France).

### Comité d'organisation des SOR

A Bataillard (Centre Léon Bérard, Lyon, France; FNCLCC, Paris, France), P Bey (Institut Curie, Paris, France), H Borges-Paninho (FNCLCC, Paris, France), L Bosquet (Centre Léon Bérard, Lyon, France; FNCLCC, Paris, France), S Brusco (FNCLCC, Paris, France), J Carretier (Centre Léon Bérard, Lyon, France; FNCLCC, Paris, France), S Debuiche (FNCLCC, Paris, France), V Delavigne (Rouen, France), E Esteves, N Fabre, F Farsi and B Fervers (Centre Léon Bérard, Lyon, France; FNCLCC, Paris, France), G Gory-Delabaere (Centre Val d’Aurelle Paul-Lamarque, Montpellier), S Guillo and AG Guy (FNCLCC, Paris, France), M Haugh and L Leichtnam-Dugarin (Centre Léon Bérard, Lyon, France; FNCLCC, Paris, France), E Luporsi (Centre Alexis Vautrin, Vandoeuvre-lés-Nancy, Nancy, France), T Philip (Centre Léon Bérard, Lyon, France; FNCLCC, Paris, France), D Ropé (FNCLCC, Paris, France), S Rousmans (Centre Léon Bérard, Lyon, France; FNCLCC, Paris, France) and S Theobald (Centre Paul Strauss, Strasbourg, France).
